# Advancing precision livestock farming in pigs through markerless pose estimation: a comparison between DeepLabCut and SLEAP

**DOI:** 10.1093/af/vfaf052

**Published:** 2025-12-22

**Authors:** Vladimir Živković, Hassan-Roland Nasser

**Affiliations:** Department of Pig Breeding, Research and Development, Institute for Animal Husbandry, Belgrade, Serbia; Digital Production Group, Agroscope, Ettenhaussen, Switzerland

**Keywords:** computer vision, DeepLabCut, precision livestock farming, SLEAP

Implications

- The development of DeepLabCut and Social LEAP Estimates Animal Poses estimates animal poses is changing how behavioral research is done by allowing precise and detailed tracking of animal movements. These tools make it possible to study complex behaviors more accurately than traditional observation methods, opening new opportunities for different research fields.
- Differences in installation, annotation, and training show that researchers should choose the tool that best matches their technical skills, project goals, and experimental setup. Selecting the appropriate framework enhances research efficiency and accessibility.
- The structured annotation methods and evaluation approaches used in both frameworks can help create more consistent and comparable data. This is important for reproducibility and collaboration between different research groups.
- Since both frameworks reach similar levels of accuracy, future work can focus more on improving usability, workflows and adding downstream tasks of interest for the community, making these tools easier to adopt and integrate into different types of research.

## Introduction

In modern agriculture, the pursuit of efficiency, sustainability, and higher welfare standards has driven rapid growth of Precision Livestock Farming (PLF). PLF integrates sensors, automation, and data analytics to continuously monitor individual animals, turning raw data into actionable insights that improve both productivity and care. In swine production, PLF tools are increasingly used to track growth, feed intake, and behavioral changes, providing early warnings of stress, illness or compromised welfare (Benjamin and Yik, 2019).

Traditional methods such as manual observation, weighing and invasive devices (e.g., RFID tags, accelerometers) are labor-intensive, disruptive and somewhat limited. Noninvasive, scalable methods applicable across diverse housing environments are therefore increasingly sought. Computer vision has emerged as a promising solution, particularly through markerless pose estimation, which extracts detailed morphological and behavioral data directly from video streams without physical contact.

Markerless pose estimation applies deep learning to detect and track anatomical landmarks (nose, ears or limbs) over time. Operating unnoticeably, it can potentially suit livestock environments where group size, crowding, and dynamic interactions challenge traditional methods. Beyond generating coordinates, these systems quantify posture, gait, and activity. While first introduced in neuroscience (Mathis et al., 2018; Nath et al., 2019; Lauer et al., 2022), enabling fine-grained studies of rodents, primates, and fish, these methods are now being adapted to livestock science, where scalability and robustness are crucial.

Two frameworks dominate the field: DeepLabCut (DLC) and SLEAP (Social LEAP Estimates Animal Poses). DLC, introduced in 2018 (Mathis et al., 2018), pioneered transfer learning for animal tracking, showing that accurate models could be trained with fewer labeled images. SLEAP, introduced in 2022 (Pereira et al., 2022), was designed for multi-animal contexts. It integrates identity-tracking modules and offers a polished graphical user interface. Unlike DLC, which grew from single-animal origins, SLEAP embedded robustness for crowded settings from the start, making it particularly suitable for commercial pig housing.

These developments enable a systematic comparison of DLC and SLEAP in swine research. Positioned within the broader PLF landscape, the two frameworks represent not just tools but innovative approaches in animal science, with the potential to transform welfare monitoring, breeding and management into pig production. In this article, we provide a comprehensive comparison between the two frameworks, both from the documentation as well as from a practitioner perspective where we test both software on a sample video dataset considering the whole pipeline from installation, to performing the actual work such as annotation and modelling to exploitation of results. The user experience and the performance of the two software were compared.

## DepLabCut and SLEAP: Background

### DeepLabCut

Mathis et al. (2018), showed that transfer learning, reusing networks pretrained on large datasets such as ImageNet (Deng et al., 2009), could achieve reliable pose estimation with just 100–200 annotated frames. This integration lowered barriers to entry and quickly established DLC as one of the leading tools for single-animal tracking in neuroscience.

Originally built on TensorFlow, DLC implemented ResNet (ResNet-50, 101, 152) backbones (He et al., 2016) and lighter alternatives such as MobileNet (Howard et al., 2017). With version 3.0, DLC transitioned to PyTorch (Lauer et al., 2022), simplifying installation, improving training speed, and enabling greater flexibility for custom networks, data augmentation, and external library integration.

DLC has grown well beyond its original scope:

- Multi-Animal DLC (MA-DLC): introduced by Lauer et al. (2022), enabling simultaneous tracking of multiple animals. Identity assignment works effectively but can fail under occlusion or dense interactions.
- Three-Dimensional Tracking: by calibrating multiple cameras, DLC can reconstructs 3D kinematics, offering motion-capture-level detail without markers (Nath et al., 2019).

With more than 3,400 citations, DLC supports a large user base across neuroscience, ethology and agriculture, providing tutorials, Slack workshops, and a growing library of pre-trained models.

### Social LEAP estimates animal poses

SLEAP was designed specifically for multi-animal contexts (Pereira et al., 2022). It supports multiple architectures, including UNet, ResNet, and EfficientNet, allowing users to optimize speed or accuracy. Its workflow is fully integrated into a graphical interface that covers annotation, skeleton definition, model training, and validation. Importantly, SLEAP offers:

- Top-Down Approaches: detect individuals first, then estimate landmarks.
- Bottom-Up Approaches: detect all landmarks and assign them to individuals.

This flexibility enables adaptation across different farms and experimental setups.

SLEAP’s key strengths include robust identity tracking and readiness for real-time monitoring (Pereira et al., 2022). Identity modules maintain consistent labeling of individuals across frames, even during occlusions or crossings, which are common in group pens. Optimizations for GPU (Graphical Processing Units) acceleration and export to TensorRT/ONNX can allow near real-time inference, which opens possibility for live welfare monitoring.

Direct comparisons of DLC and SLEAP highlight trade-offs. Scholz et al. (2025) tested both frameworks, on zebrafish videos, under varied imaging conditions, including changes in resolution and camera exposure. Results showed that:

- DLC achieved lower false negative rates when conditions matched the training data but was sensitive to resolution changes, losing accuracy when videos were down sampled or cameras differed in quality.
- SLEAP maintained more consistent error distributions across different conditions but showed a higher rate of false negatives, occasionally missing landmarks altogether.

### The use of DLC and SLEAP in pig livestock farming in the literature

**Figure 1.**
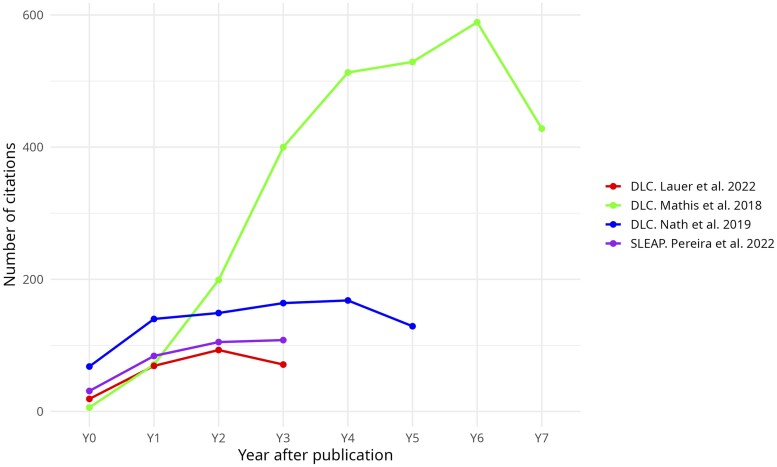
The number of citations for each framework post publication. The picture shows a wider long-term adoption of the DLC framework compared to the SLEAP framework. The last year (Y7; October 2025 at the time of writing this manuscript) shows a decrease in number of publications for all the three DLC related papers.

DLC has seen broader adoption in scientific research compared to SLEAP, primarily due to its earlier introduction and the larger, more established user community it has built over time. Figure 1 shows the citation numbers for the 3 DLC papers that contributed to the software in addition to the SLEAP papers. Given the same number of years after publication, the main DLC paper (Mathis et al., 2018) gained a wider adoption compared to SLEAP.

Gorssen et al. (2022) demonstrated DLC’s capacity to extract key phenotypic traits such as shoulder width, body length, and hip height from more than 1,500 pigs, developing a rapid pipeline with root mean square errors of just 1.8 cm compared to ground truth, underscoring the potential for high-throughput, longitudinal phenotyping at commercial scales. Zhao et al. (2023) proposed a noncontact, rapid pig body size measurement method, achieving accurate real-time results. These automated measurements were both precise and genetically and productively informative, replacing manual methods and significantly reducing labor and animal stress. Farahnakian et al. (2021) showed that DLC can handle multi-pig scenarios with strong accuracy, though performance dropped when models were transferred to barns with different lighting and flooring. This highlights the difficulty of generalizing pose estimation models across heterogeneous farm conditions. SLEAP’s identity-preservation modules may offer a complementary strength in such contexts, enabling the tracking of individuals over time and making it particularly suitable for studying social behaviors like aggression, dominance hierarchies, and space use in group pens. De Paula et al. (2024) focused on locomotion, but the framework’s structure is easily extended to group-level behavioral analysis.

Sow–piglet interactions present another important application area. Farahnakian et al. (2024) used DLC to monitor these interactions in free-farrowing systems, reliably quantifying postures, and nursing behaviors despite frequent occlusions and rapid movements. Automating the analysis of sow lying behavior, piglet activity, and nursing events represents a major improvement over manual video processing. Lameness detection also remains a major welfare and economic challenge in pig farming. De Paula et al. (2024) trained models to assess sow gait in dorsal-view setups. More recently, Liu et al. (2023) introduced the ZS-DLC-PAF (ZhangSuen-DeepLabCut-PartAffinityField) framework, which leverages skeleton-based representations for more fine-grained posture and behavior detection, offering tools to potentially identify subtle abnormalities related to health or welfare concerns.

Although this review focuses on pigs, DLC and SLEAP have been applied successfully in cattle, poultry, and sheep (Doornweerd et al., 2021; Li et al., 2023; Mahmoud, 2024). Dairy cow studies have used pose estimation to track lying behavior and detect lameness, while poultry research has analyzed keypoint prediction. These applications illustrate the flexibility of pose estimation and offer valuable insights into scaling and adapting such technologies across diverse housing systems. Despite these advances, several barriers remain before DLC and SLEAP can be routinely deployed on farms. One major issue is model generalization. Models trained in one environment often perform poorly when transferred to farms with different lighting, flooring, pen structures, or skin colors.

Another critical challenge is the annotation burden. While DLC reduces the number of required annotations compared to training models from scratch, robust applications still require thousands of labeled frames. Liu et al. (2023) used over 14,000 frames, and de Paula et al. (2024) annotated more than 5,500. This scale is often beyond the capacity of individual research groups and impractical for on-farm adoption. Active learning tools in DLC and SLEAP can prioritize informative frames, but expert input remains necessary to ensure biologically meaningful landmark definitions, which also introduces risks of inter-annotator variability.

Even with accurate models, questions remain about whether current systems meet welfare-critical thresholds. Liu et al. (2023) reported 85% node detection accuracy, while de Paula et al. (2024) achieved 0.72 average precision in locomotion models, but such levels may still be insufficient for clinical decision-making. Missing early signs of illness or distress can have serious welfare and economic consequences. Moreover, the field lacks standardization in skeletal landmark definitions, behavior annotation, and evaluation metrics, making cross-study comparisons difficult and slowing methodological convergence. Scholz et al. (2025) demonstrated the benefits of sharing annotated datasets and pre-trained models; wider adoption of this practice could standardize skeletons, reduce annotation demands, and accelerate progress.

## A Practitioner’s Comparison of DeepLabCut and SLEAP

To better understand the practical aspects of these frameworks, we conducted hands-on testing on a workstation equipped with modern GPUs (16 GB RAM). Both DeepLabCut and SLEAP were evaluated on the same multi-animal video dataset to compare usability, annotation workflow, and model performance. DLC (v3.0.0rc8) was installed via the python package manager pip with PyTorch as the training engine. SLEAP (v1.4.1) was installed using conda-managed environment, as the precompiled package with TensorFlow/Keras backend. Both frameworks were run under Linux (Ubuntu 20.04) with CUDA 11.6.

Recordings were obtained using high-definition video cameras (e.g., 1,280 × 720 pixels at 30 frames per second) under controlled farm conditions. Videos from multi-animal experiments were collected to assess user experience and the performance of both pose estimation frameworks across experimental contexts. Lighting, arena design, and background were kept constant to minimize environmental variability. For testing, one video, with six piglets, has been selected and cut (around 7,500 frames). For each framework, 100 frames were extracted. Frames were manually annotated with user-defined body parts (head, left ear, right ear, four points across the back and tail) to generate labeled datasets. Annotation was carried out using each framework’s native labeling interface: the DLC labeling GUI (Graphical User Interface) for multi-animal projects (Mathis et al., 2018; Lauer et al., 2022), and the SLEAP graphical interface for multi-animal datasets (Pereira et al., 2022). To reduce potential annotation bias, identical subsets of frames were labeled across both frameworks.

Two independent pipelines were implemented. For DLC, models were trained using the PyTorch backend, with top_down_hrnet_w32 (resnet50) network as the primary backbone. Training followed the standard DLC pipeline: frame extraction, dataset creation, training set generation, and iterative optimization of network weights (Mathis et al., 2018; Nath et al., 2019). For multi-animal experiments, the Multi-Animal DLC (MA-DLC) extension was employed (Lauer et al., 2022). For SLEAP, training was performed within the integrated GUI. Models were trained using UNet-based architecture, with hyperparameters left at recommended defaults. The video data and the two configuration files obtained from SLEAP and DLC can be found in this data repository: doi:10.5281/zenodo.17202361.

When it comes to complexity of installation process, DLC and SLEAP are different. DLC has historically relied on older versions (before 3.0.0), which often makes GPU setup tricky because of compatibility issues between the NVIDIA libraries CUDA and cuDNN. Installation usually requires carefully managed conda environments with strict version matching, and this process can be frustrating. By contrast, SLEAP was designed with ease of use in mind. It offers prebuilt installers through conda, pip, and even standalone binaries, which greatly reduces the risk of dependency conflicts. GPU support is integrated more smoothly, and the overall installation process tends to be much faster and less error prone. The difference also shows up in documentation and community support. DLC benefits from a very large user base in neuroscience and ethology, which means there are many tutorials, FAQs, and community threads (GitHub) to draw from. However, a significant portion of these discussions focus on troubleshooting installation issues, highlighting its steeper setup curve. SLEAP, conversely, has documentation that is written with accessibility in mind, offering straightforward, step-by-step installation guides.

**Figure 2.**
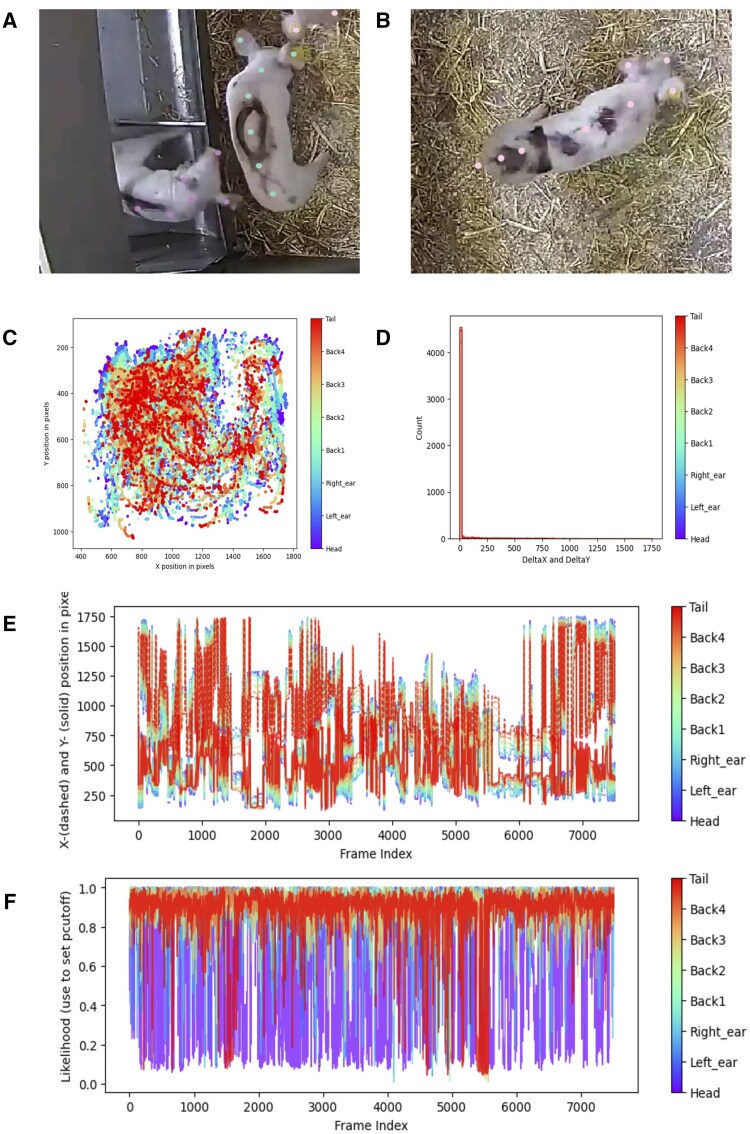
Representative graphical assets from the DLC software. Example of annotating nonoccluded parts (a) and occluded parts (b). In DLC, annotating occluded points is possible but tricky. (c–f) Outputs concern only one animal. (c) The heatmap 2D coordinates of each keypoint for the selected individual in the image space. (d) The histogram of 2D frame-to-frame displacement of each keypoint. (e) Maps the 2D coordinates of each keypoint, and (f) the likelihood of each key point per frame.

Annotation is another area where the two tools differ significantly in terms of user experience. In DLC, annotation is functional but somewhat basic. The labelling GUI allows for manual placement of keypoints, but the interface is not particularly optimized for speed or large-scale projects (Figure 2). Label management and frame navigation can become cumbersome, and handling multi-animal datasets often adds further complexity. Consequently, the author’s experience showed DLC was faster overall, as it was needed only about three hours to annotate the dataset and setup the project.

**Figure 3.**
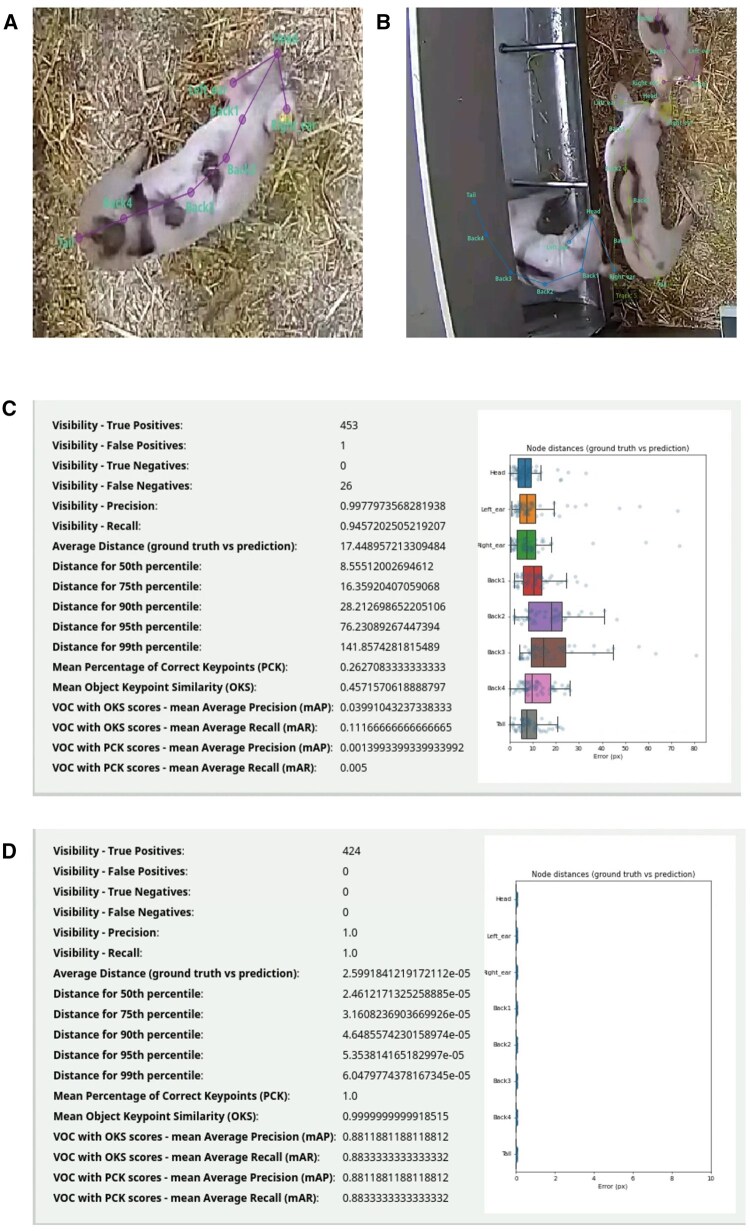
Representative graphical assets extracted from the SLEAP software. Example of annotating nonoccluded parts (a) and occluded parts (b). In SLEAP, the user inserts a complete skeleton and then adjusts the keypoints. When parts of the animal are occluded, approximate positions must still be provided for all keypoints. (c) The performance metrics centered instance model and (d) the performance metrics of the centroid model.

SLEAP, in contrast, puts much more emphasis on a modern, streamlined design. Its annotation interface feels up to date, with intuitive frame navigation and skeleton-based labelling integrated from the start (Figure 3). This skeleton-first approach reduces errors during annotation because body parts are always tied to a defined structure, preventing inconsistencies that can happen with free keypoint placement. However, we found that this came at the cost of time: annotation in SLEAP required about double the hours compared to DLC. But even so, the modern interface and reduced error rate make SLEAP particularly attractive for complex datasets or multi-animal recordings where annotation consistency is crucial.

Another example of annotation differences between the two software tools is occlusion. When part of the pig’s body is occluded, DLC does not require annotating that hidden part. In contrast, SLEAP mandates annotating all body parts, including those under occlusion, due to its skeleton-based annotation design (Figure 3).

In training and testing, DLC and SLEAP again feel quite different. DLC provides extensive manual control: one can configure the dataset, choose the backbone, and track progress through loss curves, but testing and visualization require extra steps. It performs well once the workflow is understood, though it remains less integrated. SLEAP, by contrast, keeps everything in one place. Training, evaluation, and visualization are integrated in the GUI, with defaults that work out of the box and plots that update as process goes on. From a user’s perspective, DLC often feels faster if there is previous familiarity with structure of the workflow, while SLEAP takes more time to set up but is smoother and less error-prone once the process is running, especially for multi-animal data.

DLC and SLEAP network training report different metrics, which explain the contrast in numbers. Our DLC model reached mAP@50 of 96% and mAP@[50:95] of 72%, showing strong detection even under stricter thresholds. The summary file adds that training achieved mAP = 99.1%/mAR = 99.7% with RMSE = 1.87 px, while on the test set performance was still high at mAP = 87.35%/mAR = 89.33% with RMSE of 8.53 px meaning nearly all keypoints are found and localized within a few pixels.

SLEAP presents the same performance differently: visibility precision of 99.7% (similar to DLC’s high mAP@50), average distance of 17px (the counterpart to DLC’s RMSE), and much lower OKS/PCK mAP values (0.04 or lower) due to stricter evaluation rules that penalize even small errors (Figure 3).

Taken together, both frameworks tell a consistent story: they detect almost all keypoints correctly and localize them with small pixel error and note that the apparent gap in mAP values comes from different metric definitions rather than real differences in accuracy. For easier comparison all aspects are summarized in Table 1.

**Table 1.** Comparison of DLC and SLEAP through the experiment

Aspect	DeepLabCut (DLC)	SLEAP
Version/installation	The version v3.0.0rc was used Installation via the python package manager (pip) and the system in conda PyTorch backend Installation may be tricky due to CUDA/cuDNN compatibility.Requires careful environment management.	The version v1.4.1 was used Installed via precompiled conda package (TensorFlow/Keras). Provides prebuilt installers and smoother GPU support.
Annotation experience	Functional but basic GUI for multi-animal labeling. Navigation can be inefficient when correcting mistakes. Faster overall (approx. 3 h for dataset setup).	Modern skeleton-based GUI with intuitive navigation. Reduces annotation errors because of the rigid skeleton-based annotation Took roughly double the time but ensured consistency.
Training pipeline	Uses PyTorch with top_down_hrnet_w32 (ResNet50) backbone. Workflow: frame extraction → dataset creation → training → optimization. Multi-Animal DLC extension used → Inference	UNet-based architectures trained within the SLEAP GUI using default hyperparameters. Supports centroid and top-down pipelines. frame extraction → dataset creation → training → Inference
Possibility of active learning	Possible using the ‘refining tracklets’ feature	Not included as a feature in the user interface, but theoretically possible, though not intuitively for regular users
User experience (training)	High manual control; requires extra steps for visualization. Faster if experienced, but less integrated. Computation process slower than SLEAP.	Integrated GUI with real-time plots. Slower setup but smoother and less error-prone, especially for multi-animal datasets. Computation process faster.
Performance metrics (train)	mAP@50 = 96 %, mAP@[50:95] = 72 %. Training summary: mAP = 99.1 %, mAR = 99.7 %, RMSE = 1.87 px.	Visibility precision = 99.7 %; avg. distance = 17 px; OKS/PCK mAP ≤ 0.04 (stricter evaluation).
Performance metrics (test)	mAP = 87.35 %, mAR = 89.33 %, RMSE = 8.53 px. Nearly all keypoints localized within a few pixels.	Evaluation metrics differ; lower OKS/PCK due to stricter tolerance, not necessarily lower accuracy.
Outputs from training stage	Logs from the training is aggregated in .csv and .f5 files together with model weights snapshots (and best model), training configs and terminal log.	Performance metrics (.csv, .h5); training config; network evaluation (.csv, .h5).
Outputs from evaluation phase	A csv file contining the evaluation result on the training and test set Pictures of predictions showing ground truth versus model predictions	The model performance using the centered and centroid models (Figure 3c and d)
Outputs from inference phase	Video with tracking; heatmaps of body parts position (Figure 2c), histogram of 2D frame-to-frame movement for each animal (Figure 2d); likelihood of positions (Figure 2f) and 2D position for each body part in each frame per animal (Figure 2e).	.slp project file; rendered video; inference analysis (.csv, .h5).
Overall summary	Requires more technical expertise but provides high control and excellent accuracy. Annotation faster but less structured. Denser and more intuitive output allowing easier interpretation and exploration of data.	Easier installation and integrated workflow. Annotation slower but more consistent. Performance broadly comparable under different metric schemes.

### Future directions

While applications of DLC and SLEAP show clear potential for pigs, scaling these methods to commercial farms requires addressing some possible improvements. Several priorities stand out for advancing markerless pose estimation in PLF.

In addition to benchmark datasets, a major step forward will be the creation of standardized annotation protocols. Currently, each research group defines its own skeletons and annotation protocols, making results difficult to compare. A standardized set of annotated videos covering varied housing systems, lighting, and age groups would enable direct benchmarking of DLC, SLEAP and emerging tools. Shared evaluation metrics, beyond DLC’s Mean Absolute Error and SLEAP’s precision scores, would further harmonize comparisons. Open repositories, modeled on initiatives like ImageNet (Deng et al., 2009), would also lower barriers and promote reproducibility. Integrating complementary data streams could increase robustness. Developing frameworks for real-time fusion of these modalities could give us more reliable welfare assessments.

New machine learning architectures also promise to improve performance. Lightweight networks and transformers offer efficiency gains, while approaches such as GANPose (Wang et al., 2023) enhance robustness under noisy conditions. Incorporating temporal information across frames could improve identity stability and reduce errors. Currently, researchers are limited to use the nontime-based models for detection and pose estimation and some are obliged to add an additional step to the pipeline to classify behavior or make action recognition by using Long short-term memory (LSTM) (Chen et al., 2020; Gan et al., 2021; Wang et al., 2023). Ultimately, pose estimation may evolve toward dynamic behavior recognition, classifying sequences such as lying, fighting, or limping, rather than detecting static landmarks alone.

Perhaps most crucially, systems must be validated under commercial farm conditions. Unlike controlled barns, large-scale farms involve variable pen layouts, poor lighting, high stocking densities and environmental noise. Real-time responsiveness is vital, as detecting events like sow distress during farrowing requires immediate alerts.

## Conclusion

By enabling the automated tracking of anatomical landmarks without invasive markers or wearable devices, frameworks such as DLC and SLEAP open new possibilities for monitoring pigs in real time, at scale and in farm-realistic conditions. Their growing adoption reflects both the technological maturity of deep learning-based computer vision and the increasing demand for objective, high-frequency welfare and productivity data in livestock systems.

This comparison has highlighted the complementary strengths of DLC and SLEAP. DLC excels in precision, flexibility, and integration with advanced machine learning workflows, making it particularly suitable for detailed morphometric ­analyses and controlled research contexts. SLEAP offers robust identity preservation, real-time performance, and user-friendly interfaces that offer more practicality for animal scientists.

Applications in pig research already span body-size measurement, multi-pig tracking, farrowing behavior, and locomotion monitoring, with new methods such as skeleton extraction.

By embracing collaboration and open science, the livestock research community can ensure that pose estimation becomes not just a research tool but a cornerstone of data-driven pig production, enhancing both productivity and animal welfare.

## Author Biographies

**Vladimir Živković** is a researcher at Institute for Animal Husbandry, Department of pig breeding, research and development, Belgrade, Serbia. His work focuses on studying pigs’ nutrition and behavior using modern methods and tools. He holds a PhD degree in Animal Science from the University of Belgrade, Faculty of ­Agriculture. His research interests include ­computer vision and its applications for automating farm observation and classifying behavior of pigs.

**Hassan-Roland Nasser** is a researcher at Agroscope in the Digital Production group. His work focuses on applying artificial intelligence to challenges in agriculture, particularly in livestock and smart farming. Before joining Agroscope, he lectured in data science at French and Lebanese universities. He holds a PhD in Computational Neuroscience from INRIA and a degree in Biomedical Engineering from the University of ­Technology of Compiègne. His research interests include computer vision and its applications for automating agricultural observation and image analysis.
